# Expression Pattern of Inflammatory Response Genes and Their Regulatory MicroRNAs in Bovine Oviductal Cells in Response to *Lipopolysaccharide*: Implication for Early Embryonic Development

**DOI:** 10.1371/journal.pone.0119388

**Published:** 2015-03-12

**Authors:** Sally Ibrahim, Dessie Salilew-Wondim, Franca Rings, Michael Hoelker, Christiane Neuhoff, Ernst Tholen, Christian Looft, Karl Schellander, Dawit Tesfaye

**Affiliations:** Institute of Animal Science, Animal Breeding and Husbandry Group, University of Bonn, Bonn, Germany; Huazhong Agricultural University, CHINA

## Abstract

In the present study, we used an *in vitro* model to investigate the response of the oviduct with respect to inflammatory mediators and their regulatory microRNAs in case of bacterial infection and subsequent association with embryo survival. For this, we conducted two experiments. In the first experiment, cultured primary bovine oviductal cells (BOEC) were challenged with *lipopolysaccharide* (*LPS*) for 24h and the temporal expression pattern of inflammatory mediators and their regulatory microRNAs were measured at 0, 3, 6, 12, 24 and 48h after *LPS* treatment. Intriguingly, the temporal patterns of all miRNAs except miR-21 were significantly up-regulated at 6h after *LPS* treatment. Whereas, we observed significant overexpression of pro-inflammatory mediators as tumor necrosis factor alpha (TNFα) and interleukin-1 beta (IL1β) after *LPS* challenge for 24h. On the other hand, the expression level of essential elements like oviductal glycoprotein 1 (OVGP1) and insulin-like growth factor 2 (IGF2) was significantly decreased in challenged groups compared with control. Moreover, miR-155, miR-146a, miR-223, miR-21, miR-16 and miR-215 have shown a clear suppression in challenged group after *LPS* treatment. In the 2nd experiment there were four groups of blastocysts produced, namely embryo+*LPS* free media, embryo+*LPS*, BOEC+embryo and BOEC+embryo+*LPS*. The suboptimal oviduct environment due to LPS challenge is found to have a significant influence on the expression of inflammatory response genes (TNFα and CSF1), stress response genes (SOD and CAT), mitochondrial activity, reactive oxygen species (ROS) accumulation and apoptotic level either in cultured or co-cultured blastocysts. Collectively, *LPS* challenge led to aberrant changes in oviductal transcriptome profile, which could lead to a suboptimal environment for embryo development.

## Introduction

In cattle, bacterial contamination of the uterine lumen is ubiquitous after parturition, and up to 40% of animals develop pelvic inflammatory disease (PID) and 20% have endometritis [1,2]. Infection of the endometrium with *Escherichia coli* (*E*. *coli*) precedes infection by other pathogenic bacteria and viruses, which paves the way for other pathogens to cause endometrial damage and disrupt ovarian cycles, including extended luteal phases [3]. The cost of female reproductive disorders and the associated infertility was estimated to be $650 million per annum in the United States [4,5].

Oviduct is the female genital organ at which oocyte maturation, sperm capacitation, fertilization and transport of gametes and embryos is occurring (maturation, capacitation etc do not occur in other organ), [6] and the disturbance of oviduct function due to pathogenic infection could result early embryonic loses and infertility. The surface of the oviduct and other female reproductive systems are lined with mucosa which can act as a physical barrier against the outside environment and participates in both innate and acquired immune defence [7,8]. The mucus faces the challenge of different antigenic/ inflammatory stimuli arises during mating or parturition [9,10]. The effect of endometritis on ovarian function is a well documented fact in which higher and moderate level of *LPS* concentrations were found in follicular fluid collected from post partum cows with clinical and subclinical endometritis, respectively compared with the normal ones [9]. As an intermediate organ between the uterus and the ovary, there is a higher chance that the oviduct will be under the influence of *LPS*. Despite the mucosal role in immune defence of the genital tract, little is known about the mechanism of bovine oviduct epithelial cell activation by pathogens or about the receptors and secondary mediators involved in this response. It is well established that Toll-like receptors (TLRs) have important roles in detecting pathogens and in initiating inflammatory responses that subsequently prime specific adaptive immune responses during infection [11]. It has also been recognized that dysregulation of this process is a hallmark of inflammatory and autoimmune diseases [12]. TLR4, in association with accessory molecules MD-2 and CD14 are required for Gram-negative bacterial *LPS* detection. Binding of TLR4 to *LPS* initiates a cascade of signaling events that evokes the production of cytokines and pro-inflammatory mediators [13]. Although the functional role of TLR4 in the intact endometrium and oviduct of human, mouse and in the intact endometrium of bovine has been explored, less is known about its role in bovine oviduct [10,14,15].

To date, the early embryonic death in bovine is a poorly understood phenomenon and this is attributed to the paradoxical biological and immunological cellular activities involved in this process. It was estimated that up to 40% of total embryonic losses occur between days 0−7 of pregnancy in cattle [16–18]. In addition, in laboratory and domestic farm animals, it is becoming increasingly clear that the oviducts play a critical role in the development of the zygote and during the early cleavage stages‒a phase that embraces the transition from maternal to embryonic regulation of the genome. In some manner yet to be explained, the exposure to the oviduct milieu acts to promote an integrated unfolding and expression of the embryonic gene programme [19].

Here, we report the establishment and optimization of an *in vitro* model, as a tool to test whether TLR4 and its accessory molecules (CD14/MyD88) are essential for the response to minimum dose of *LPS* by bovine oviduct epithelial cells. In addition to decipher the molecular & cellular changes mediated by *LPS* on oviduct function and to investigate subsequent effects on early embryonic development in bovine will enable us to draw the association between oviductal infection and early embryonic death in bovine. The aim of the current study was (I) to address the changes in inflammatory response mRNAs and their targeting miRNAs expression profile in oviductal epithelial cells after *LPS* challenge, (II) to investigate the subsequent effect of challenged oviductal cells on co-cultured embryos viability. For this, we have selected candidate inflammatory and stress response genes namely: tumor necrosis factor alpha (TNFα), interleukin-1 beta (IL1β), TNF receptor associated factor 6 (TRAF6), insulin-like growth factor 2 (IGF2), oviductal glycoprotein 1 (OVGP1), caspase 3, apoptosis-related cysteine peptidase (CASP3), transforming growth factor, beta 1 (TGFβ1), superoxide dismutase 1 (SOD) and glutathione peroxidase 4 (GPX4), which could have a crucial role in oviductal function as well as immune response against bacterial infection. Moreover, we have investigated their potential regulatory miRNAs including miR-16, miR-21, miR-223, miR-215, miR-146a and miR-155 in primary bovine oviductal epithelial cells exposed to minimum dose of *LPS*.

## Material and Methods

All chemicals and reagents for *in vitro* culture were purchased from Sigma Aldrich unless otherwise stated. Plastic dishes, four-well plates, and tubes were obtained from Nunc (Thermo Scientific). All chemicals for reverse transcription were acquired from Invitrogen (Life Technologies). The whole experiment was replicated at least three independent biological replicates each done in quadruplicate.

The use of *in vitro* produced bovine embryos for this experiment was approved by the Animal Welfare committee of the University of Bonn with proposition number 84-02.05.20.12.075. But all the other experiments were conducted using oviductal cells and ovaries collected as by-products of the local abattoirs (Bernhard Frenken GmbH Vieh- und Fleischhandel Schlachthof Düren and EFM Euskirchener Fleisch MarktGmbH) which are slaughtering animals for local meat consumption.

## Experiment 1: Effect of in vitro *LPS* challenge on bovine oviductal epithelial cells

### Bovine oviductal epithelial cell culture (BOEC)

The collection and culture of oviductal cells were performed as described previously [20]. Briefly, oviductal tissue ipsilateral to corpus luteum (CL) was collected from five cows after slaughter and transported to the lab in phosphate buffer saline without Ca^2+^/Mg^2+^ (PBS^−^) supplemented with 2% Penicillin-Streptomycin (Gibco, Karlsruhe; Germany). Upon arrival in the laboratory, the oviducts were rinsed in 70% ethanol followed by three times washing in PBS^−^. The oviduct ampulla was gently squeezed in a stripping motion with forceps to obtain epithelial cells. After repeated pipetted, the cell suspension was passed twice through a 23 gauge (G) syringe needle and then incubated for 30 min to allow the fibroblast cells to adhere to the culture dish. The cells were then washed with fresh culture medium and allowed to sediment for 25 min. Then cells were seeded in a 24‒well culture plate in a concentration of 10^6^ cells per well in 800 μl culture medium. An aliquot of the cell suspension was used for cell count. The proportion of alive cells prior seeding was assessed by Trypan blue staining (Gibco, Karlsruhe; Germany). The cells were cultured at 38°C in a humidified atmosphere with 5% CO2 in air. Culture medium was changed every 2‒3 days, until the cells attained 85‒90% confluency.

### Cell culture treatment

After cells reached confluency, one group of cells were challenged with *LPS* and untreated groups were used as a control. The cells were challenged with arachidonic acid (AA, 100 μM), (Sigma, Munich; Germany) and with 0.5 μg/ml of Ultra-pure *LPS* (InvivoGen, San Diego; USA), the *LPS* concentrations at a level found in the uterine lumen during sub-clinical infected animals [9]. The cells were harvested at one time point (after 24h) from *LPS* challenge as well as at different time points (0, 3, 6, 12, 24 and 48h) from *LPS* challenge to observe temporal expression pattern of miRNAs. The supernatant and harvested cells were kept in −80°C for molecular analysis. In addition, the cell culture supernatant was collected for measurement of TNFα and prostaglandins (PGE_2_ & PGF_2α_) by using specific bovine ELISA kits. The absence of immune cells in the oviduct epithelial cell cultures was confirmed by PCR for the CD45 pan-leukocyte marker as previously described [21,22] (data not shown). Cell viability was assessed by using WST-1 Cell Proliferation Assay Kit (Cayman Chemical, Michigan; USA).

### RNA isolation and cDNA synthesis

Total RNA was isolated from the cells using the miRNeasy Mini kit (Qiagen, Hilden; Germany) according to manufacture’s protocol. In order to remove possible contaminations of genomic DNA, the extracted RNA was subjected to on-column DNA digestion by using RNase free DNase set on-column (Qiagen, Hilden; Germany). The cDNA for gene expression analysis was synthesised from the isolated total RNA using SuperScript II (Invitrogen, CA; USA). Briefly, maximum volume of 10 μl RNA from each replicate of the treatment was co-incubated with 0.5 μl of 100 μM Oligo(dT)_15_ (Promega, WI; USA) and 0.5 μl of Random Primer (Promega, WI; USA) at 70°C for 3 min then chilled on ice for 2 min. Reverse transcription was performed in a 20 μl mixture of 4 μl 5x first strand buffer, 2 μl 0.1 M DTT, 1 μl 10 mM dNTP, 0.7 μl Superscript II reverse transcriptase (Invitrogen, CA; USA), 0.3 μl RNasin (Promega, WI; USA) and RNase free water was added to the RNA mixture in a PCR strip and run in a thermocycler programmed at 42°C, 90 minutes; 75°C 15 minutes and hold at 4°C. The cDNA was synthesis for small RNA from the isolated total RNA using the miScript II RT kit (Qiagen, Hilden; Germany) following manufacturer’s protocols. Briefly, 2 μl of total RNA samples were mixed with reverse-transcription master, which composed of 4 μl 5x miScript HiSpec, 2 μl 10x Nucleics Mix, 2 μl miScript Reverse Transcriptase Mix in a 20 μl reaction volume. Reaction incubation was performed at 37°C for 60 min followed by heating at 95°C for 5 min to inactivate miScript Reverse Transcriptase. Depending on the amount of total RNA used for RT-PCR, the resulting cDNA samples were diluted before use as a template for miRNA quantitative real-time PCR assay.

### Quantitative real-time PCR analysis of selected candidate genes

Gene specific primers were designed using Primer3 Program version 4.0 (http://primer3.ut.ee/) [23]. Details of primers are described in (Table 1). The specificity of each primer amplicon was evaluated by sequencing the PCR products using GenomeLab GeXP Genetic Analysis System (Beckman Coulter).Quantitative real-time PCR of mRNAs was performed in a StepOnePlus Real-Time PCR System (Applied Biosystems, Foster City; CA), using SYBR Green/ ROX Mix (Qiagen, Hilden; Germany), with the following program: 95°C for 3 min, 40 cycles at 95°C for 15 s, 60°C for 1 min and 95°C for 1 min. Melting curve was evaluated at the end of the run to observe the specificity of the amplification. The data was analyzed by the comparative threshold cycle (∆Ct) method and normalization was performed using geometric of GAPDH, β-actin (ACTB) and 18S.

**Table 1:** List of primers that were used for semi-quantitative PCR analysis of target genes.

Gene name	Accession no.	Primer sequence (5′→3′)	Annealing temperature (°C)
CAT	NM_001035386	F: agccagaagagaaaccctca	53
		R: ctgcctctccatttgcatta	
TGFβ1	NM_000660	F: cacgtggagctgtaccagaa	55
		R: gcgaaagccctctatttcct	
IGF1	NM_001077828	F: ttgcacttcagaagcaatgg	54
		R: actggagagcatccaccaac	
INOS	NM_001076799	F: tgttcagctgtgccttcaac	55
		R: aaagcgcagaactgagggta	
CASP3	NM_001077840	F: tgccactgtatcagggaaca	52
		R: tgctcagcacaaacatcaca	
TRAF6	NM_001034661	F: CCCAGGCTGTTCAGACTTTA	53
		R: CATACATGCTCTGGGTTTCC	
CD14	NM_174008	F: tatcgtggacaacaggaggt	54
		R: gcgtagcgctagatattgga	
TLR4	NM_174198	F: agagccacttctggtcacag	55
		R: taaagctcaggtccagcatc	
MYD88	NM_001014382	F: cctctcatctgcctttttga	53
		R: gccccagaaagaaagacttc	
CD45	NM_001206523	F: caaagagcccaggaagtaca	51
		R: gttgatctccacaatcaca	
IL1β	NM_174093	F: ccttgggtatcaaggacaag	53
		R: cgatttgagaagtgctgatg	
SOD	NM_174615	F: ccttgggtatcaaggacaag	50
		R: cgatttgagaagtgctgatg	
TNFα	NM_173966	F: cttccacccccttgttcct	55
		R: aggcgatctcccttctcca	
IGF2	XM_005227270	F: gccctgctggagacttactg	54
		R: ggtgactcttggcctctctg	
OVGP1	NM_001080216	F: ctctgcacccacctggtatt	54
		R: GCGATCACTGAACTGACGAA	
GAPDH	NM_001034034	F: acccagaagactgtggatgg	57
		R: acgcctgcttcaccacctt	
18S	NR_036642	F: cgcagctaggaataatggaa	53
		R: tctgatcgtcttcgaacctc	
ACTB	NM_173979	F: ggcattcacgaaactacctt	53
		R: caatccacacggagtacttg	
NFκB1	NM_001076409	F: aatttgggaaggatttggag	55
		R: Ctgtcgtttcctttgcactt	
CTSB	NM_174031	F: tccccatagacgaactgtgt	55
		R: Tctcagatctgtcccactcc	
LIF	NM_173931	F: agaccagaaggtcctcaacc	55
		R: Acagcccagcttcttcttct	
CSF1	XM_005204070	F: ctttgtcactgggatggaag	55
		R: tttcctgatccagagagtgc	

### Quantitative real-time PCR analysis of selected candidate miRNAs

The differential expressed genes in BOEC due to *LPS* challenge were uploaded into miRNA prediction tools namely: DIANA-microT v3.0 (http://diana.cslab.ece.ntua.gr/microT/) and miRecords (http://mirecords.biolead.org/). Then we filtered the miRNA hits on the basis their potential relevance for physiological function and immune response of oviduct at least in four different search algorithms, and thus, miR-16, miR-21, miR-223, miR-215, miR-146a and miR-155 were identified as a potential miRNAs targeting our genes of interest. Interestingly, miR-16, miR-21, miR-223 and miR-215 were reported by [24], where these miRNAs are differentially expressed between healthy and sub-clinical endometritis cows, but miR-146a and miR-155 are indicated to be endotoxin-responsive genes [25,26]. Sequence specific miRNA primers were used to quantify the candidate miRNAs with the corresponding mature sequences listed in (Table 2). In addition, a functional annotation analysis was performed using DAVID Bioinformatics Resource (http://david.abcc.ncifcrf.gov/).

**Table 2:** The list of miRNAs with the corresponding mature sequences amplified using quantitative real time PCR.

MiR name	Accession no.	Mature miRNA sequence (5′→3′)
bta-miR-16b	MIMAT0003525	UAGCAGCACGUAAAUAUUGGC
bta-miR-21	MIMAT0003528	UAGCUUAUCAGACUGAUGUUGACU
bta-miR-223	MIMAT0009270	UGUCAGUUUGUCAAAUACCCCA
bta-miR-215	MIMAT0003797	AUGACCUAUGAAUUGACAGACA
bta-miR-146a	MIMAT0009236	UGAGAACUGAAUUCCAUAGGUUGU
bta-miR-155	MIMAT0009241	UUAAUGCUAAUCGUGAUAGGGGU

Quantitative real-time PCR of these miRNAs was performed using sequence specific miRNA primer sets and miScript SYBR Green PCR kit (Qiagen, Hilden; Germany). Prior to real-time PCR profiling, 200 μl RNase-free water was added to each 20 μl reverse-transcription reaction. A PCR master mix was prepared using 2 μl of cDNA template, 12.5 μl of 2x QuantiTect SYBR Green PCR Master mix, 2.5 μl of 10x miScript Universal primers, 2.5 μl of 10x miScript Primer Assay. RNase-free water was added to final volume 25 μl. Quantitative real-time PCR was performed in a StepOnePlus Real-Time PCR System (Applied Biosystems, Foster City; CA). The thermal cycling conditions were initial activation step 15 min at 95°C followed by 3-step cycling; denaturation 15 s at 94°C, annealing 30 s at 55°C and extension 30 s at 70°C (40 cycles). Melting curve analysis was constructed to verify the presence of specific amplification. The data was analysed by the comparative threshold cycle method and normalization was done using geometric mean of the two endogenous controls [5S and U6 snRNA (Exiqon, Vedbaek; Denmark)].

### ELISA for TNFα and PGs (PGE_2_ and PGF_2α_) concentrations in the culture media

The concentration of TNFα and PGs were measured in the cell culture supernatant from *LPS* challenge and control cell groups at different time points using commercially available bovine specific TNFα (Bethyl Laboratories, Montgomery; USA) and PGE_2_ &PGF_2α_ (Oxford Biomedical Research, Oxford; USA), respectively, following the manufacturer’s instructions. The optical density (OD) value was detected using ELISA microplate reader (Labequip Ltd, Ontario; Canada) at 450 nm wavelengths for TNFα and at 650 nm for PGs using an ELISA microplate reader (Labequip Ltd, Ontario; Canada).

### Immunoblotting

Proteins from lysates of cultured cells were normalized to 1 mg/ml using a NanoDrop ND-8000 spectrophotometer and separated (10 μg/lane) using gradient gel 4‒18% (vol/vol) SDS-PAGE. After electrophoresis, proteins were transferred to nitrocellulose membrane (Whatman- Protran, Rodgau; Germany). Membranes were incubated with antibodies for TNFα (1:400), (LifeSpan Biosciences, Toll Free; North America), OVGP1 (1:200), and TGFβ1 (1:200), (Santa Cruz Biotechnology, CA; USA) separately for the same membrane by using stripping buffer (mild stripping) according to Abcam’s protocol (http://www.abcam.com/ps/pdf/protocols/Stripping%20for%20reprobing.pdf). Protein loading was evaluated and normalized by examining GAPDH protein levels using a GAPDH antibody (Santa Cruz Biotechnology, CA; USA). Densitometric quantification of immunoreactive bands was carried out using Quantity One analysis software (Bio-Rad, Munich; Germany).

## Experiment 2: Effect of *LPS* challenge of BOEC on embryo development and quality

### *In vitro* production of embryos

Bovine ovaries were collected from local slaughterhouse and transported to the laboratory in 0.9% physiological saline solution 37°C within 1‒3 h of slaughter. Ovaries were dipped in 70% ethanol then washed 2‒3 times in PBS^−^. Cumulus oocyte complexes (COCs) were then aspirated from antral follicles (2‒8 mm in diameter), only COCs with a homogenous cytoplasm and surrounded by at least three layers of compact cumulus cells were used for *in vitro* maturation, in a group of 50 in modified TCM199 culture media supplemented with 4.4 mM HEPES, 33.9 mM NaCHO_3_, 0.2 mM sodium pyruvate, 50 mg/ml gentamicin, 10 μl/ml FSH (Folltropin, Vetrepharm) and 12% ECS. After maturation, COCs were co-incubated with concentration of 2×10^6^ sperms/ml for *in vitro* fertilization in F-TALP as indicated previously [27]. Subsequently, presumptive zygotes were denuded by repeated pipetting and transferred to SOF culture medium [28,29] supplemented with 10% ECS. Thereafter, presumptive zygotes were transferred into wells containing 400 μl of SOF medium in four-well dishes (Thermo Fisher Scientific, Roskilde; Denmark) alone or with a BOEC (isolation and seeding mentioned before at experiment (1) of material and methods), twenty-five presumptive zygotes (n = 25‒30/group) were allocated according to the experimental design. Each group of sample namely; embryo+*LPS* free media, embryo+*LPS*, BOEC+embryo and BOEC+embryo+*LPS*, under mineral oil at 38.7°C until the blastocyst stage. The maturation, fertilization and cultural procedures were performed under 20% oxygen level. Then blastocysts were kept at −80°C for further analysis.

### RNA isolation, cDNA synthesise and real-time-PCR of selected genes from different embryo groups

In order to investigate the expression of some candidate genes related to inflammation, growth factor, apoptosis, marker for embryo quality & competence, and stress response in bovine blastocyst cultured either in SOF media or co-culture with BOEC with or without *LPS*. For this, total RNA was isolated from three independent biological replicates from each group of blastocysts, using a picopure RNA isolation kit (Arcturs, CA; USA) according to methods recommended by the manufacturer. Purified RNA was transcribed into cDNA immediately using First Strand cDNA Synthesis Kit (Thermo scientific, Schwerte; Germany). Then real-time PCR was performed in a StepOnePlus Real-Time PCR System (Applied Biosystems, Foster City; CA), using SYBR Green/ ROX Mix (Qiagen, Hilden; Germany). The thermocycler program was: 95°C for 3 min, 40 cycles at 95°C for 15 s, 60°C for 1 min and 95°C for 1 min. The data was analyzed by the comparative threshold cycle (∆Ct) method and normalization was done using geometric mean of the GAPDH and β-actin (ACTB).

### Measurement of ROS

ROS level in blastocyst stage embryos in experiment 2 was performed using the H_2_DCFDA fluorescent probe (6-carboxy- 2’,7’-dichlorodihydrofluorescin diacetate), (Life technologies, Darmstadt; Germany). Fifteen blastocysts from each group were incubated with 400 μl of 5 μM H_2_DCFDA for 20 minutes in dark at 37°C. Then embryos were washed twice in PBS and the images were captured immediately under inverted microscope (Leica DM IRB, Germany) using green fluorescence filter.

### Analysis of mitochondrial distribution

We visualized the pattern of mitochondrial distribution in bovine blastocyst on day 7 cultured either in normal SOF or co-cultured with BOEC, challenged with or without *LPS*, using MitoTracker Red CMXRos (Invitrogen, Darmstadt; Germany). Ten blastocysts from each group were incubated with 200nM of MitoTracker red dye for 45 minutes followed by twice washing with PBS^-^ and fixed with 4% formaldehyde in 4°C for overnight. Fixed specimens were mounted on the slide with VECTASHIELD Mounting Medium with DAPI (Vector Laboratories, Burlingame; CA). The stained embryos were examined under a confocal laser scanning microscope, CLSM LSM-780 (Carl Zeiss, Germany), at 40 x magnification.

### Detection of DNA fragmentation by TUNEL assay

In situ cell death was detected using Tunel assay (Roche Mannheim; Germany), as described by Paula-Lopes and Hansen [29,30]. Briefly, after embryos were fixed and permeabilized, all specimens were incubated in micro-drops of the TUNEL Kit containing 10% of the enzymatic solution (deoxinucleotidil terminal transferase enzyme) with 90% of the marking solution (2´-deoxyuridine 5´-triphosphate-dUTP+fluorescein isothiocyanate-conjugated-FITC) for 1h in a humid chamber at 37°C in the dark. Whereas, a positive control was performed by treating samples with 1 IU/μL of DNase (Promega, WI; USA) and negative control was incubated in micro-drops containing only marking solution. After washing, control and experimental samples were stained with Hoechst 33342 (Sigma, Munich; Germany) and mounted in glycerol on histological slides and observed under a fluorescence microscope. Nuclei with green fluorescence (FITC) were considered TUNEL positive (fragmented DNA). Hoechst 33342 stained all healthy and apoptotic cells.

### Data analysis

Statistical analysis of expression data was performed using Student’s t test. The values shown in graphs are presented as the mean ± standard deviation (SD) of at least three independent experiments each done in quadruplicate, *p-values* < 0.05 were considered statistically significant. GraphPad Prism 5.0 was used for data plotting.

## Results

### Effect of *LPS* on viability of oviduct epithelial cells

**Fig 1:**
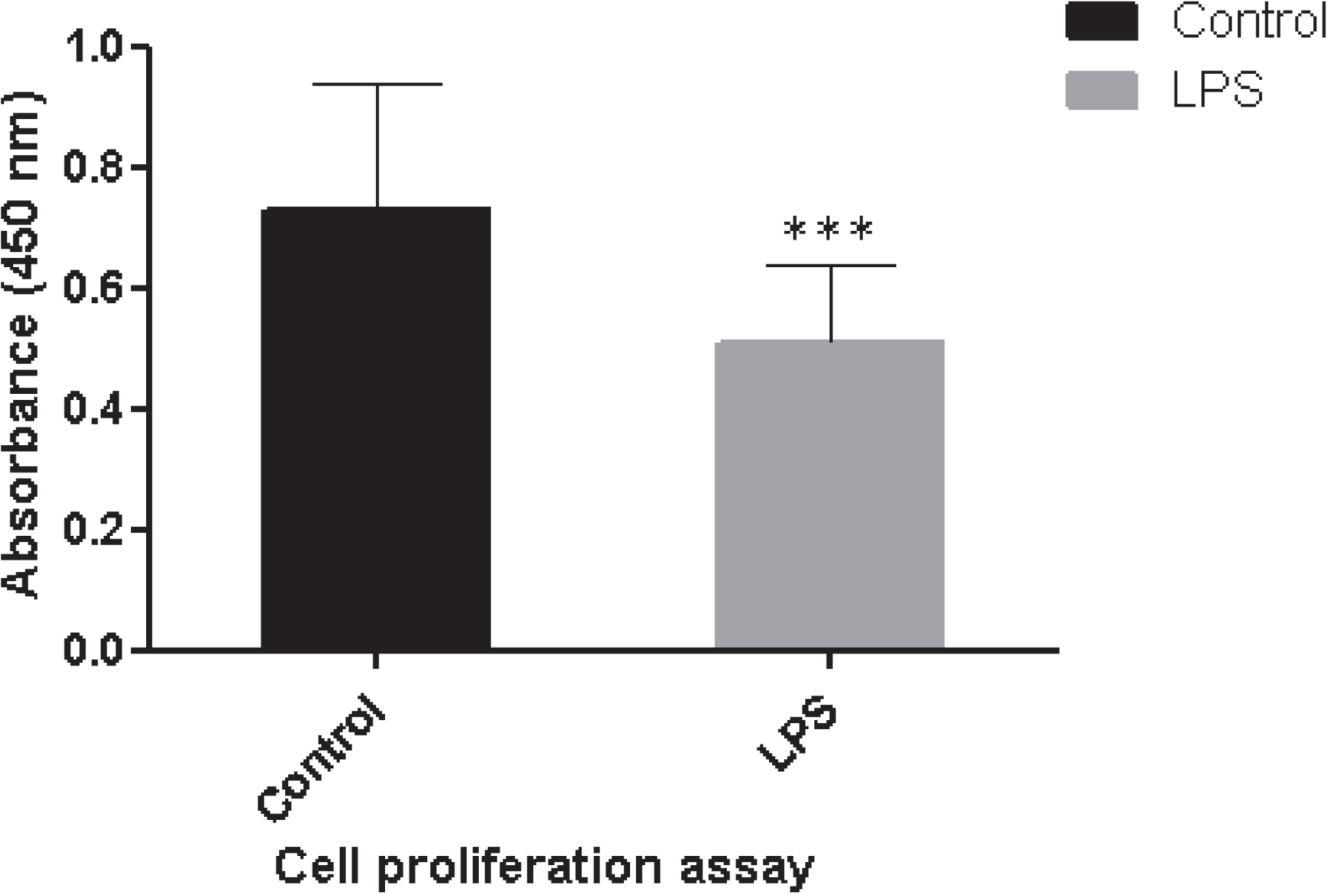
The viability of oviduct epithelial cells 24 hours after *LPS* challenge. ***: statistical significant at *p <* 0.001.

The oviduct epithelial cells viability was determined after the cells were challenge with minimal dose of *LPS* for 24h. We found the cells viability were significantly higher (*p <* 0.001) in control group compared with the challenged group (Fig. 1).

### Changes in expression of genes associated with inflammation and immunological response of BOEC after *LPS* challenge

**Fig 2:**
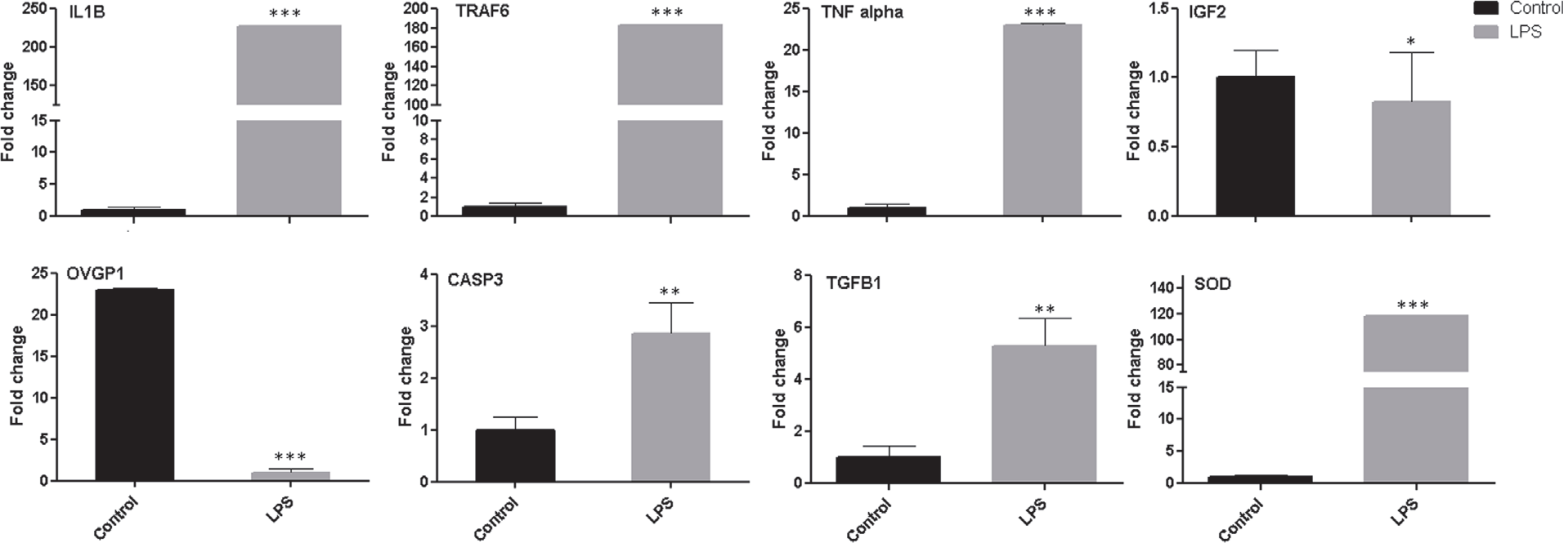
Real-time PCR analysis of candidate inflammatory response genes in oviduct epithelial cells after *LPS* challenge. Stars represent statistical significance level at *: *p <* 0.05, **: *p <* 0.01, ***: *p* < 0.001.

**Fig 3:**
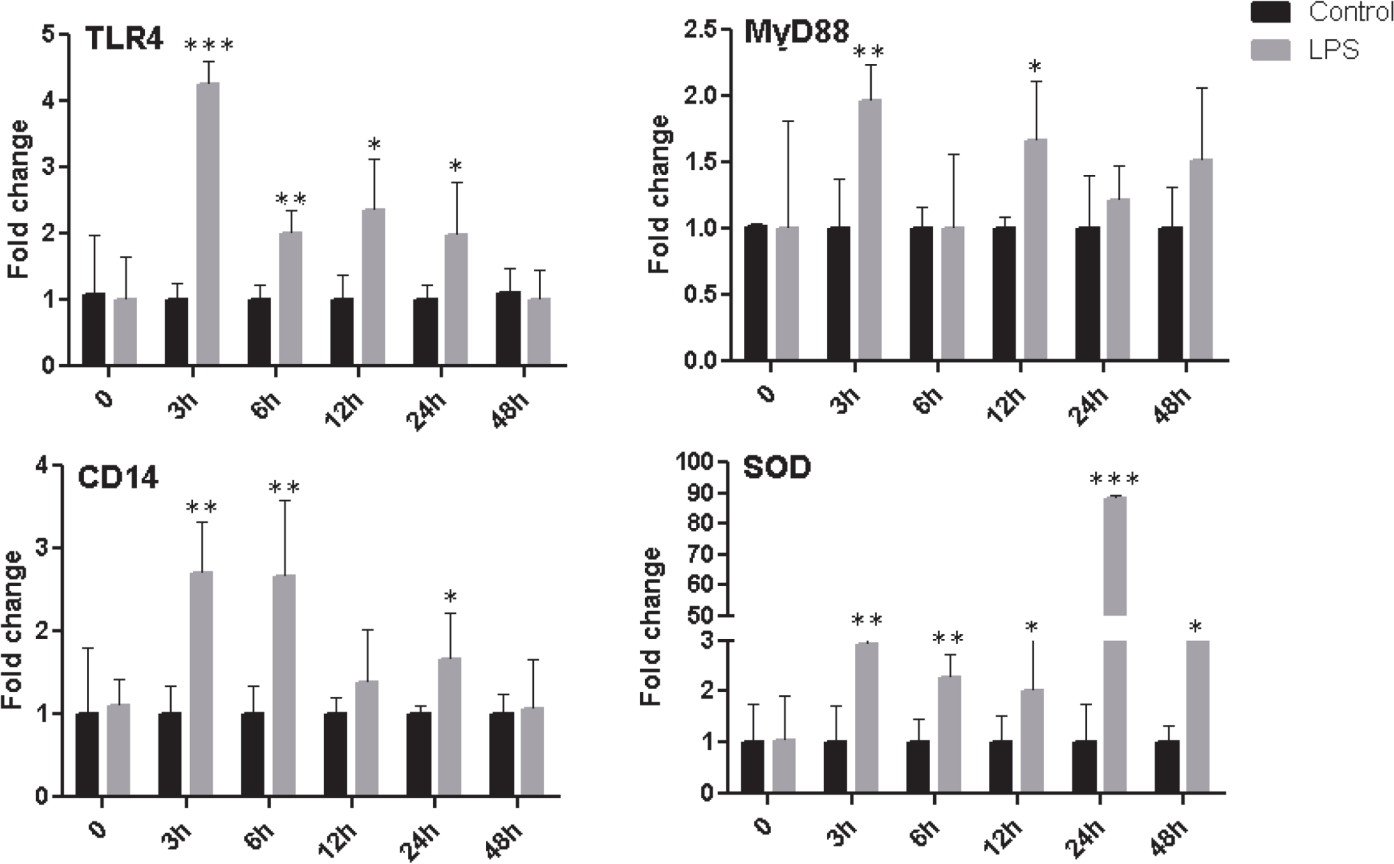
Pattern of immune and stress response genes in BOEC at different time points after *LPS*. Stars represent statistical significance level at *; *p <* 0.05, **; *p <* 0.01, ***; *p <* 0.001 mean statistical difference.

The relative abundance of IL1β, TRAF6, TNFα, CASP3, TGFβ1 and SOD was increased significantly (0.001 ≤ p ≤0.01) and the expression level of OVGP1 and IGF2 was decreased significantly in challenged group compared with control (Fig. 2). Furthermore, stimulation of primary BOEC with minimum dose of *LPS* significantly increased the expression of TLR4 and its accessory molecules (CD14/MyD88). Moreover, the expression level of stress response genes SOD was significantly up-regulated (Fig. 3), whereas the level of GPX4 was reduced after *LPS* challenge at 24h (data not shown). These results revealed that minimum dose of *LPS* can have a profound effect on transcriptome profile of primary bovine oviduct epithelial cells.

### Temporal pattern of miRNAs potentially targeting inflammatory response genes

**Fig 4:**
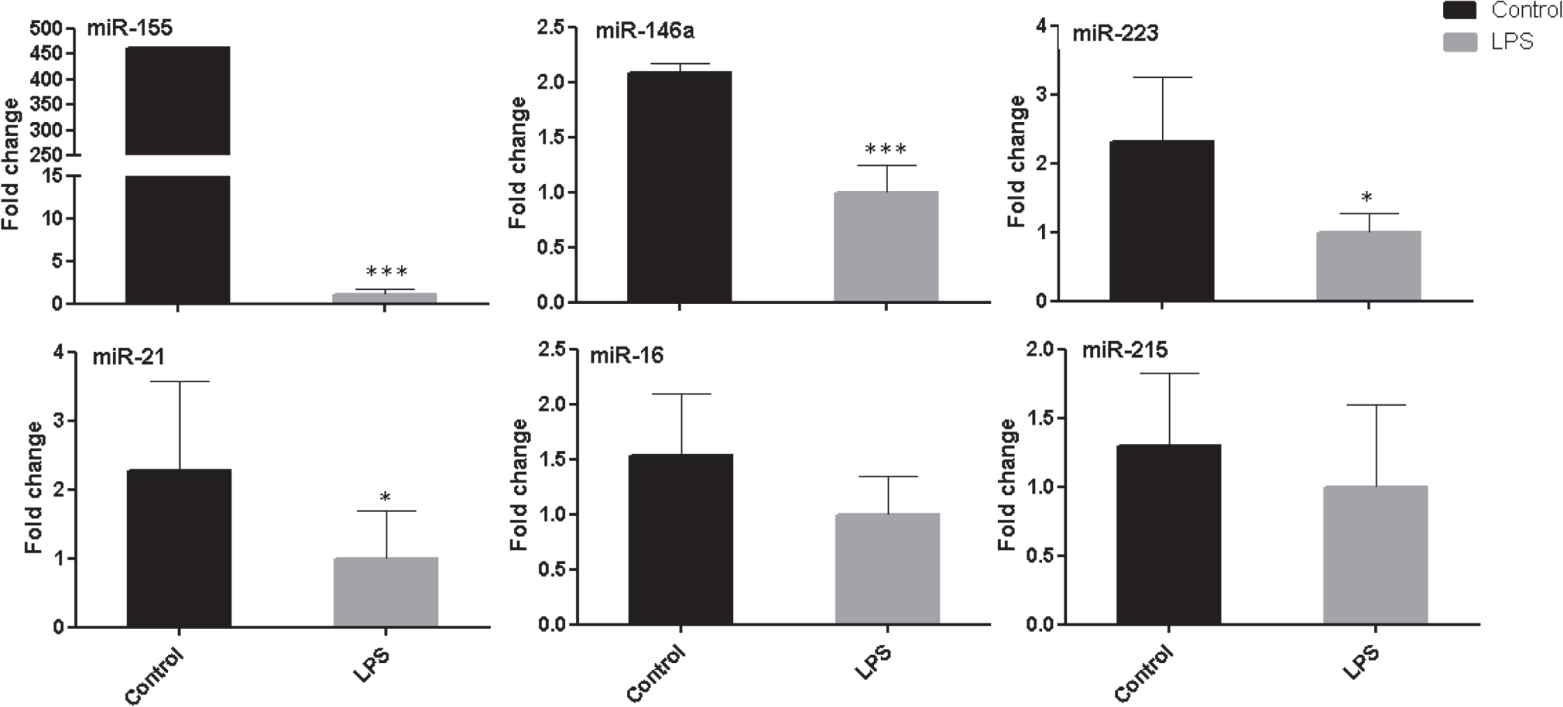
The relative abundance of selected regulatory miRNAs in BOEC after challenge by *LPS*. Stars represent statistical significance level at *; *p* < 0.05, ***; *p* < 0.001 indicate statistical difference.

We identified the potential regulatory miRNAs (miR-155, miR-146a, miR-223, miR-21, miR-16 and miR-215) targeting the candidate genes in oviductal epithelial cells, using bioinformatics tools. Then we checked alignment between seed region and 3´ UTR of selected candidate genes (S1 Fig.). Furthermore, we observed the dynamics pattern of microRNAs expression level in BOEC after *LPS* challenge at different time points (0, 3, 6, 12, 24 and 48h). Surprisingly, all miRNAs except miR-21 were significantly increased at 6h after *LPS* treatment. The expression level of some miRNAs was found to show a reciprocal pattern to their target genes (TRAF6, IL1β, TGFβ1 and TNFα), whereas the expression level of some miRNAs were found to have a similar pattern to their target genes (IGF2, OVGP1 and INOS), (S2‒S4 Figs.). The overall results showed that miR-155, miR-146a, miR-223, miR-21, miR-16 and miR-215 have shown a clear inhibition in challenged group after BOEC treated with *LPS* for 24h (Fig. 4).

### Protein level in primary BOEC in response to *LPS*

**Fig 5:**
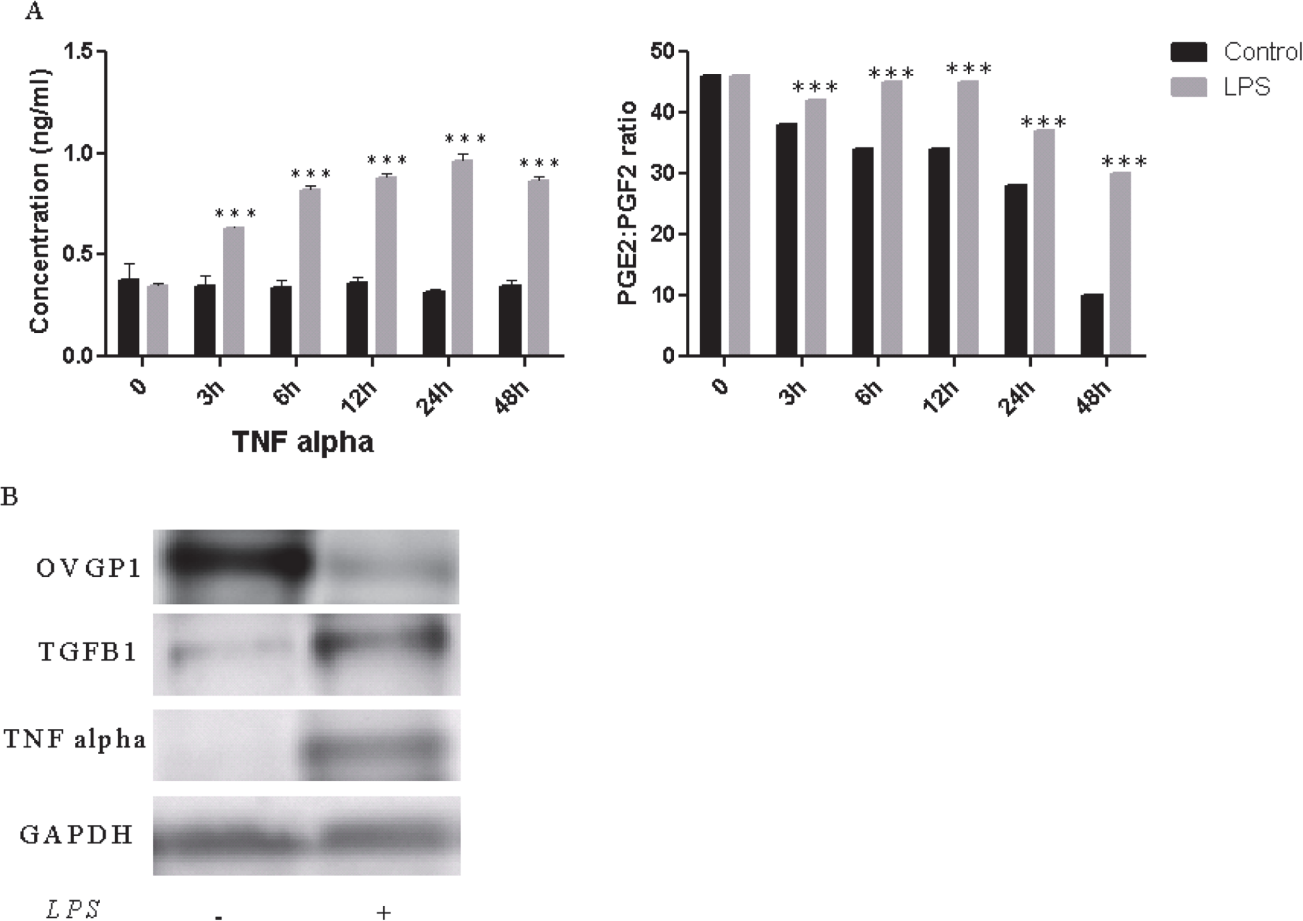
Protein expression analysis in BOEC after 24 hours *LPS* challenge. Enzyme-linked immunosorbent assay (ELISA) of TNFα and prostaglandin E_2_ (PGE_2_) to prostaglandin F_2α_ (PGF_2α_) ratio in cell culture supernatant (A). The level of OVGP1, TGFβ1 and TNFα immunoreactive proteins in oviduct epithelial cells (B). Stars represent statistical significance level at ***; *p <* 0.001.

We have determined the level of TNFα and PGE_2_/PGF_2α_ ratio in cell culture supernatant at different time points post-*LPS* treatment. TNFα level was significantly increased in *LPS* treated group compared with untreated control and the PGE_2_/PGF_2α_ ratio was significantly higher in challenged group (Fig. 5A). The OVGP1 immunoreactive protein was lower after *LPS* challenge compared with untreated controls. On the other hand, as evidenced by a clear band, TGFβ1 and TNFα were found to be higher in treated group compared with the control (Fig. 5B).

### Effect of *LPS* treated BOEC on co-cultured embryos

The percentage of cleavage rate of cultured or co-cultured embryos was not affected significantly among groups namely; embryo+*LPS* free media (83.8%), embryo+*LPS* (85.4%), BOEC+embryo (86.2%) and BOEC+embryo+*LPS* (88%). On the other hand, in 5 replicates, the average blastocyst rate of oocytes in *LPS* challenged groups with or without BOEC was significantly lower (15.7±7.78, 25.6±6.84, respectively), than those unchallenged groups (22.6±10.98, 37.5±9.47, respectively), (Table 3).

**Table 3:** Developmental rates of presumptive zygotes which were cultured either in SOF or co-cultured with BOEC, with/without *LPS*.

Cultured / co-cultured	Matured oocytes	Embryonic development		
	(n)	Cleavage rate		Blastocyst rate
		**n**	%	**Mean±SD**
SOF	**517**	**433**	**83.75**	**22.59±10.98** ^**a**^
SOF+LPS	**520**	**444**	**85.38**	**155.66±7.78** ^**b**^
BOEC+ embryo	**522**	**450**	**86.20**	**37.51±9.47** ^**c**^
BOEC+ embryo+LPS	**525**	**462**	**88.00**	**25.60±6.84** ^**d**^

Cleavage rate per oocyte is given as percentage, and blastocyst rate per oocyte is given as mean of replicates ± SD.

^a,b,c,d^ Values with different superscripts within columns differ significantly.

### Alterations in relative abundance of mRNA in co-cultured bovine blastocyst

**Fig 6:**
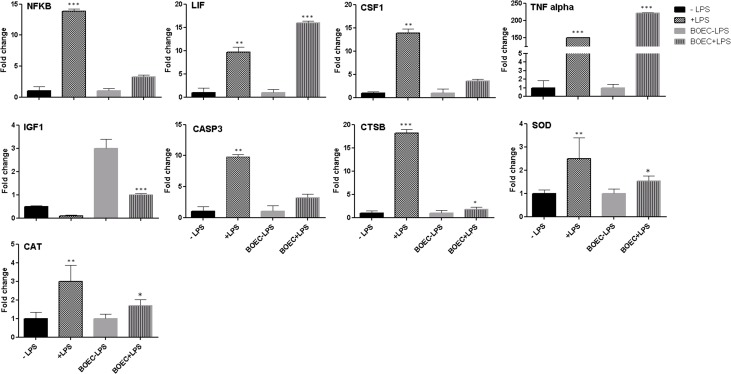
Quantitative expression analysis of genes in bovine blastocysts produced after culture or co-culture with/without *LPS*. Genes related to inflammation (NFκB, LIF, CSF1 and TNFα), Growth factor (IGF1), apoptosis (CASP3), marker for embryo quality & competence (CTSB) and stress response (SOD and CAT). Stars represent statistical significance level at *; *p <* 0.05, **; *p <* 0.01, ***; *p* < 0.001.

Here we quantified some candidate genes related to inflammation (NFκB, LIF, CSF1 and TNFα), growth factor (IGF1), apoptosis (CASP3), marker for embryo quality & competence (CTSB) and stress response (SOD and CAT), in bovine blastocyst cultured either in SOF media or co-culture with BOEC with or without *LPS*. The inflammatory response genes (NFκB, LIF, CSF1 and TNFα) were significantly increased in challenged embryos with *LPS*. Notably, stress response genes as SOD and CAT were significantly higher expressed in *LPS* treated groups compared with untreated. Furthermore, embryos quality gene (CTSB) and apoptotic gene (CASP3) were up-regulated in embryos challenged with *LPS*. Only IGF1 was up-regulated in untreated groups (Fig. 6).

### Mitochondrial distribution and ROS accumulation in co-cultured bovine blastocyst

**Fig 7:**
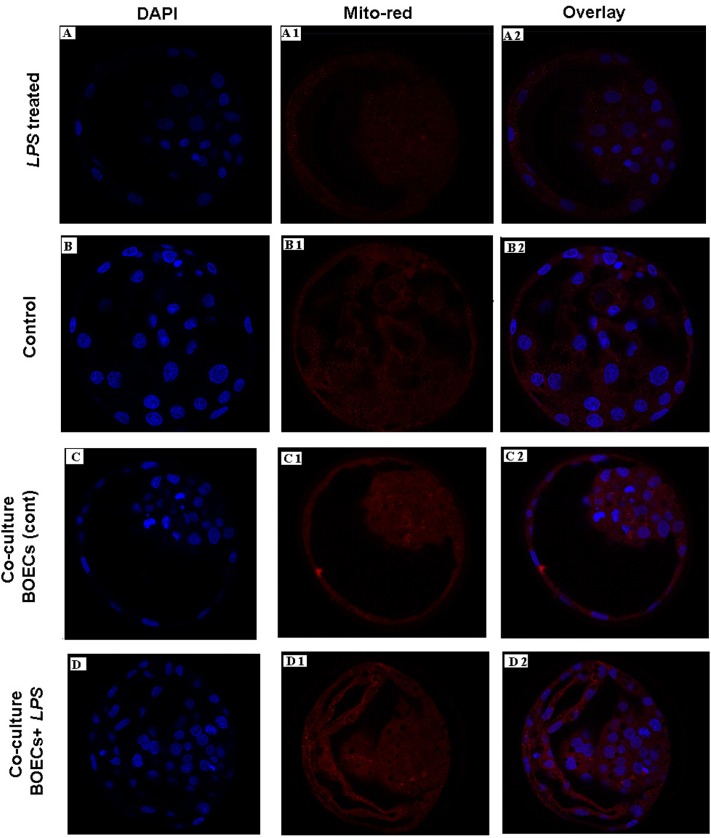
Pattern of active mitochondria as detected by MitoTracker red in bovine blastocyst produced after *LPS*. A, B, C & D showed stained nuclei using DAPI with blue fluorescent, A1, B1, C1& D1 showed mitochondria stained with MitoTracker red and A2, B2, C2 & D2 indicated a merged images. Original magnification 40×

In order to gain insight whether *LPS* could alter the distribution pattern of mitochondrial in bovine blastocyst, day 7 bovine blastocysts were produced either from SOF media or co-culture with BOEC with/without *LPS*, then incubated with MitoTracker red. We observed inadequate distribution of mitochondria and decreased mitochondrial functional efficiency, which was associated with higher ROS production in *LPS* treated groups compared with untreated controls. So we suggest that *LPS* has a deteriorated effect on mitochondria, which could compromise further embryonic development (Fig. 7 and S5 Fig.).

### Detection and quantification of apoptosis

**Fig 8:**
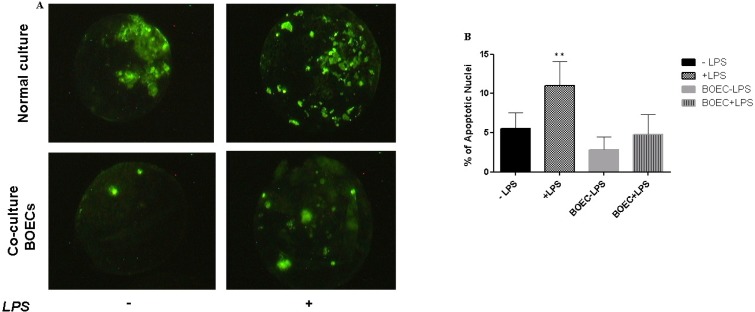
Detection of apoptotic nuclei in bovine blastocysts using TUNEL assay. Representative images of TUNEL assay to assess the level of apoptosis in blastocyst, which produced either from SOF media or co-culture with BOEC with/without *LPS* (A). The number of individual cells that were TUNEL positive was counted in each blastocyst and is represented as the average number of cells that are TUNEL positive per blastocyst (B). **; *p <* 0.01 indicates statistical difference.

Distributions of the TUNEL-positive nuclei were higher in *LPS* challenged compared with unchallenged groups (Fig. 8A). In addition to control embryos and embryos co-cultured with BOEC in the absence of *LPS* displayed very few apoptotic nuclei per embryo (5.6% and 2.8%, respectively). In contrast, blastocysts cultured or co-cultured with BOEC in the presence of *LPS* displayed a significant increase in the percentage of apoptotic nuclei per embryo (11.01% and 4.81%, respectively), (Fig. 8B). Therefore, *LPS* induces apoptosis in preimplantation embryos.

## Discussion

The oviduct is a sterile milieu in its nature but it is readily contaminated with pathogens via uterus, peritoneal cavity and follicular fluid [9,19,31]. For this, the oviduct should be equipped with an efficient and strictly controlled immune system that would maintain optimal conditions for fertilization and early embryo development. Local immune responses, regulated by the secretions of epithelial cells, form a part of the mucosal innate immunity, recently are termed “epimmunome” [32]. Despite extensive studies shown the negative effect of clinical or sub-clinical endometritis on bovine fertility with respect to molecular genetic aspects of uterine tissue in *in vivo* or *in vitro* models, the functional understanding of bacterial infection on oviductal function has remained elusive.

In the current study, the *in vitro* approach has been used to challenge bovine oviductal epithelial cells with minimum dose of *LPS* (0.5 μg/ml), to elucidate the effect of oviductal infection on expression of inflammation related genes and their regulatory miRNAs, and subsequent influence on embryo development and quality. Here we have evidenced that BOEC immediately recognized low LPS dose through TLR4 and its accessory molecules (MyD88 and CD14), which displayed a clear dynamic pattern at different time points post *LPS* stimulation. In addition to stimulate TLR4 and its downstream genes CD14, MyD88, nuclear factor kappa B (NFκB), IL1β and TNFα expression, *LPS* switched off PGF_2α_ production thus lead to increase PGE_2_/PGF_2α_ ratio. This increased PGE_2_ production resulted in proliferation of infected epithelial cells [33].

*LPS* treatment blocked oviductal function by suppression the expression of OVGP1 and IGF2; these genes similar to components of the maternal environment that are necessary for optimal embryonic development, increased blastocyst cell number and birth of a healthy calf [34,35]. In contrast, pro-inflammatory mediators such as TNF, IL1β, TGFβ1, apoptotic gene (CASP3) and stress response genes (SOD & GPX4) were up-regulated in challenged cells [36]. Taken together, these results suggest the existence of an early signaling system to respond to infection in the BOEC.

Accumulative evidences suggest that alterations in the expression of pro-inflammatory mediators seem to be responsible for inappropriate tissue regeneration, embryo implantation failure and other reproductive disorders [37,38]. Also, resolution of the inflammatory response is necessary for reparative mechanisms to take place [39]. It has become clear that miRNAs are instrumental players in the arena of mammalian inflammatory responses. So far, the role miRNAs in bovine oviduct against bacterial infection especially Gram negative bacteria, are unknown. Therefore, we checked some selected miRNAs, which are targeting most of expressed genes in BOEC in response to *LPS* stimulation. Herein, all miRNAs were decreased after *LPS* challenge. These results are similar to a recent report [40] that mentioned miRNA regulations in mammalian host cells challenged with various microbial pathogens, such as *let-7* were significant decreased in patients with *H*.*pylori* infection. Moreover, we observed dynamic pattern of miRNAs expression at different time points. Intriguingly, all selected miRNAs except miR-21 reached to peak at 6h after *LPS* stimulation. So it seems that certain miRNA functions may only be revealed at a specific concentration of an environmental trigger. furthermore, it might hold true for miRNA controlled pathways that are related to immune response [41].

Cytokines are important immunoregulatory mediators at the mammalian maternal-fetal interface. An improper balance of the pro- and anti-inflammatory cytokines (Th1 and Th2, respectively) is known to play a role in the intrauterine infection pathway [42,43]. Here, we checked embryonic development and quality in terms of cleavage rate, blastocyst rate, mitochondrial activity, ROS accumulation and apoptosis, respectively after *LPS* treatment. No significant differences were observed in cleavage rate among groups, but blastocyst rate was obviously decreased in challenged groups. These results are consistent with reports from a previously literatures [18,44,45], which showed that exposure of embryos to improper surrounding environment lead to accumulation of free radicals. Thus, resulting in lower embryo quality, survival and a delay in embryonic development.

In the present study, we found that NFκB, LIF, CSF1, TNFα were over-expressed in blastocysts produced in the presence of *LPS* challenge. Aberrant expression of these inflammatory cytokine and increased NFκB expression are some of the molecular factors that contribute to immune response disorders [46]. Notably, *LPS* showed modulation the expression of different cytokines like TNFα and growth factors like CSF1 [37]. In addition, *LPS* potentiated the release of reactive oxygen through TNFα-induced ROS production, which is known to activate NF-κB [47]. Beside the pro-inflammatory actions of TNFα, recently it was observed that the release of TNFα is associated with an increased oxidative stress [48,49] and it serves a role of ROS as second messenger to activate signaling pathways and lead to alterations in gene expression [50,51].

A recent study by [52] suggested that mitochondrial dynamics are an important constituent of cellular quality control and function. Moreover, mitochondrial ROS are important for modulating immunoreactions as part of the innate immune system through NFκB [53,54]. Furthermore, maintaining mitochondrial functions with respect to energy production and apoptosis is crucial for cellular quality and development [55]. Similarly, here we demonstrated significant alterations in mitochondrial distribution patterns in embryos challenged with *LPS*. Moreover, these alterations were associated with higher ROS production in *LPS* treated groups. Also, we examined expression of some stress response genes in blastocyst as SOD and CAT. The produced blastocyst in *LPS* treated groups showed higher abundance of SOD and CAT and it was accompanied by higher ROS generation. So we suggested *LPS* induced a remarkable increase in SOD and CAT mRNA levels, which were insufficient to scavenge the whole produced ROS, whereas *LPS* and cytokines could act synergistically to evoke more ROS [56].

Apoptosis is known to be associated with the quality and viability of mammalian embryos at preimplantation stages and it may more likely occur because of suboptimal conditions [57–59]. In the current study, *LPS* elicited a series of signal transduction events that evoke a plethora of numerous biochemical mediators, including cytokines (TNFα and CSF1) and toxic free radicals. Successful pregnancy requires a delicate balance between pro-inflammatory (Th1) and anti-inflammatory molecules (Th2), to maintain maternal immune system integrity, while preventing rejection of the embryo [60]. Therefore, the disturbance of these mediators showed an inhibitory effect on cell growth or proliferation and enhanced apoptosis in *LPS* treated groups. In agreement with previous studies [61,62], we found that CTSB and CASP3 expression increased in blastocysts challenged with *LPS* and this was associated with inferior embryos quality. Furthermore, we observed a clear suppression of IGF1 expression in groups challenged with *LPS* and this could be related to increased apoptosis and decreased blastocyst quality. Similarly, the previous studies demonstrated that the perturbed IGF signalling pathway within the oviduct affects embryo development and blastocyst cell number [8,35].

**Fig 9:**
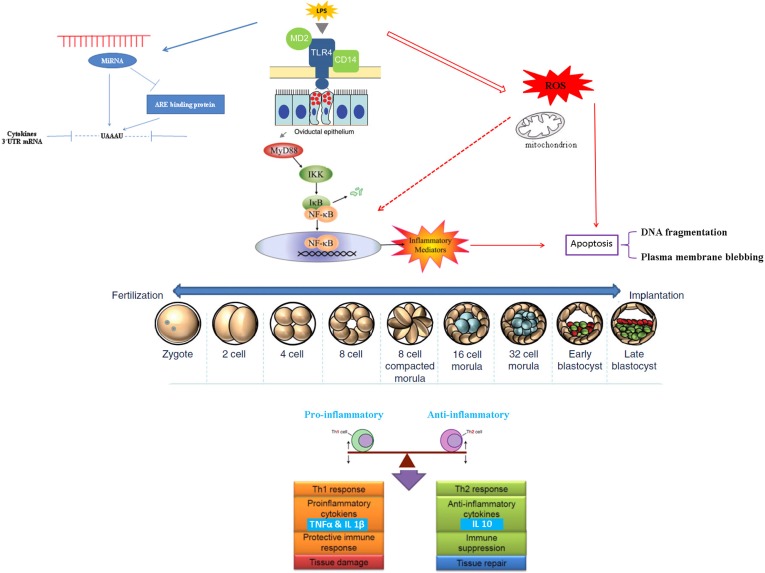
Hypothetical presentation of bovine oviduct response to *LPS* and subsequent influence on early embryonic development. Hypothetical embryo images are taken from [63].

Taken together the previous mentioned mechanisms could be implicated in female infertility and early embryonic death, we illustrated it through a schematic drawing model (Fig. 9). These findings shed a new light on relevance of inflammatory condition induced by *LPS* in oviduct milieu and subsequent early embryo development. It indicated a balance among immune mediators, mother and embryo that could act dependently and synergistically, and is one of the most elegant and fascinating interactions in first cross-talk, which takes place in oviduct between mother and embryo to initiate and maintain the embryonic development and subsequent implantation process. Meanwhile, disturbance of that delicate balance between pro-inflammatory mediators (Th1) and anti-inflammatory mediators (Th2) may be reflected on dynamic function of mitochondrial through over-production of ROS and subsequently increased apoptotic cells during early embryonic development.

## Supporting Information

S1 Fig The potential regulatory miRNAs and their alignment with the binding sites of genes 3´ UTR. (TIF)

S2 Fig Expression profile of miR-146a and its target genes (TRAF6 and IL1β). **RT-PCR of miR-155 and its target genes (IL1β, CASP3 and IGF2) in BOEC after *LPS* challenge for 48hr.** Both miRNAs and their target genes showed different dynamic pattern at different time points, where peak of both miRNAs was at 6h after *LPS* stimulation then gradually decreased.
(TIF)

S3 Fig Real-time PCR of miR-16 and its target genes (TNFα and IGF2), miR-223 and its target gene (IGF2), and miR-215 and its target gene (INOS) in BOEC after *LPS* challenge for 48hr. All miRNAs reached their peaks at 6h after *LPS* stimulation. On the other hand, some genes revealed the same trend and/or reciprocal of miRNAs.
(TIF)

S4 Fig Pattern of miR-21 expression profiling and its target genes (TNFα, TGFβ1, OVGP1 and IGF2) in BOEC after *LPS* challenge for 48hr. MiR-21 reached to peak at 12h then gradually reduced post *LPS* challenge. Both OVGP1 and IGF2 shown peaks only in untreated groups. In contrast, TNF and TGFβ1 provide clear peaks in challenged groups.
(TIF)

S5 Fig ROS generation was detected by fluorescent probe H_2_DCFDA in bovine blastocyst on day 7. ROS production in embryo co-cultured with BOEC without or with *LPS*, (A & B) respectively. ROS production in bovine blastocysts which were cultured in SOF media without or with *LPS*, (C & D) respectively. Scale bars represent 100 μm.
(TIF)
