# Supplementary figures and images for: Expression Pattern of Inflammatory Response Genes and Their Regulatory MicroRNAs in Bovine Oviductal Cells in Response to Lipopolysaccharide: Implication for Early Embryonic Development
Source: PLoS One. 2015 Mar 12;10(3):e0119388. doi: 10.1371/journal.pone.0119388 (PMC4357424; doi:10.1371/journal.pone.0119388)

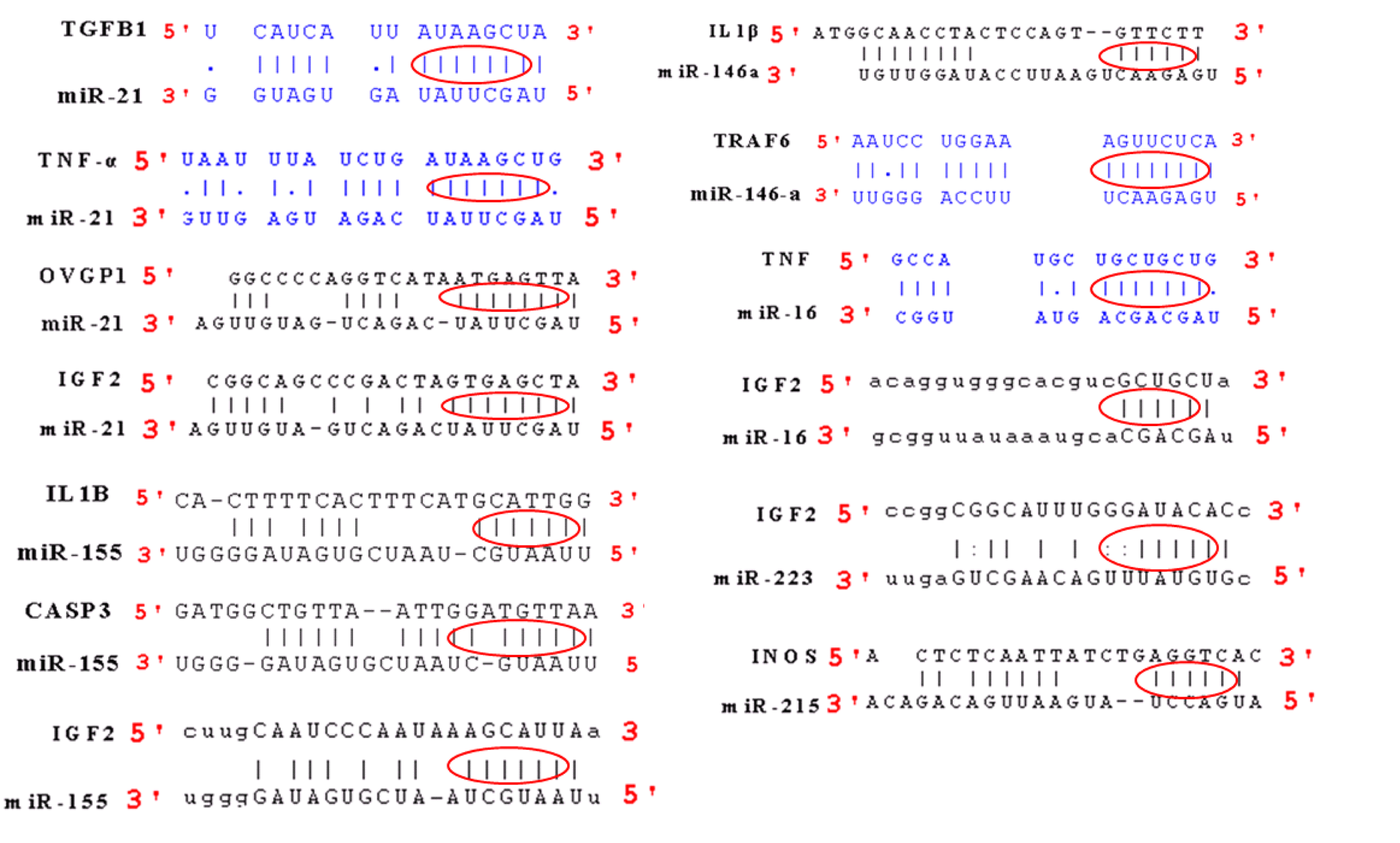

Supplement: S1 Fig — (TIF) [file pone.0119388.s001.tif]

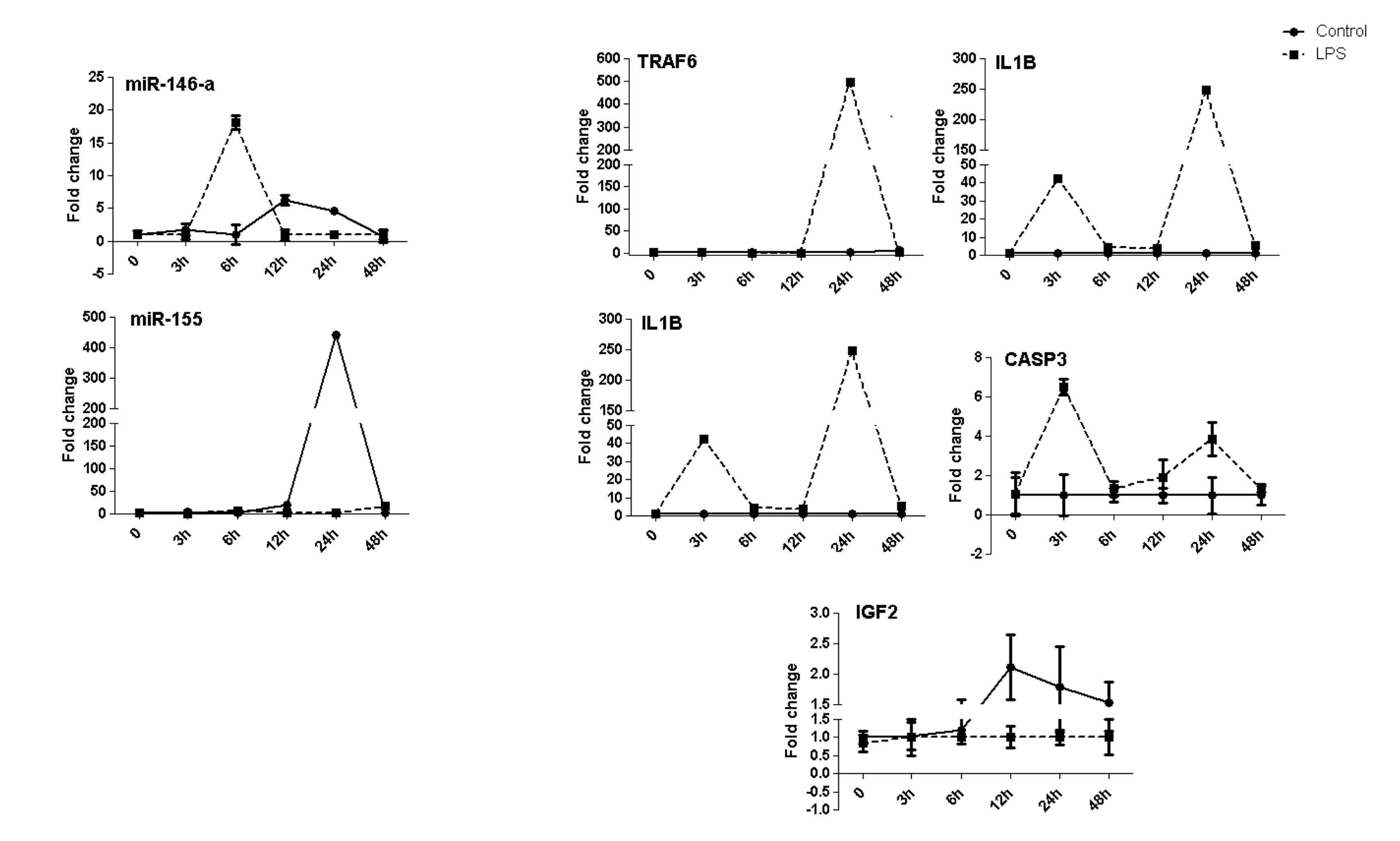

Supplement: S2 Fig — RT-PCR of miR-155 and its target genes (IL1β, CASP3 and IGF2) in BOEC after LPS challenge for 48hr. Both miRNAs and their target genes showed different dynamic pattern at different time points, where peak of both miRNAs was at 6h after LPS stimulation then gradually decreased. (TIF) [file pone.0119388.s002.tif]

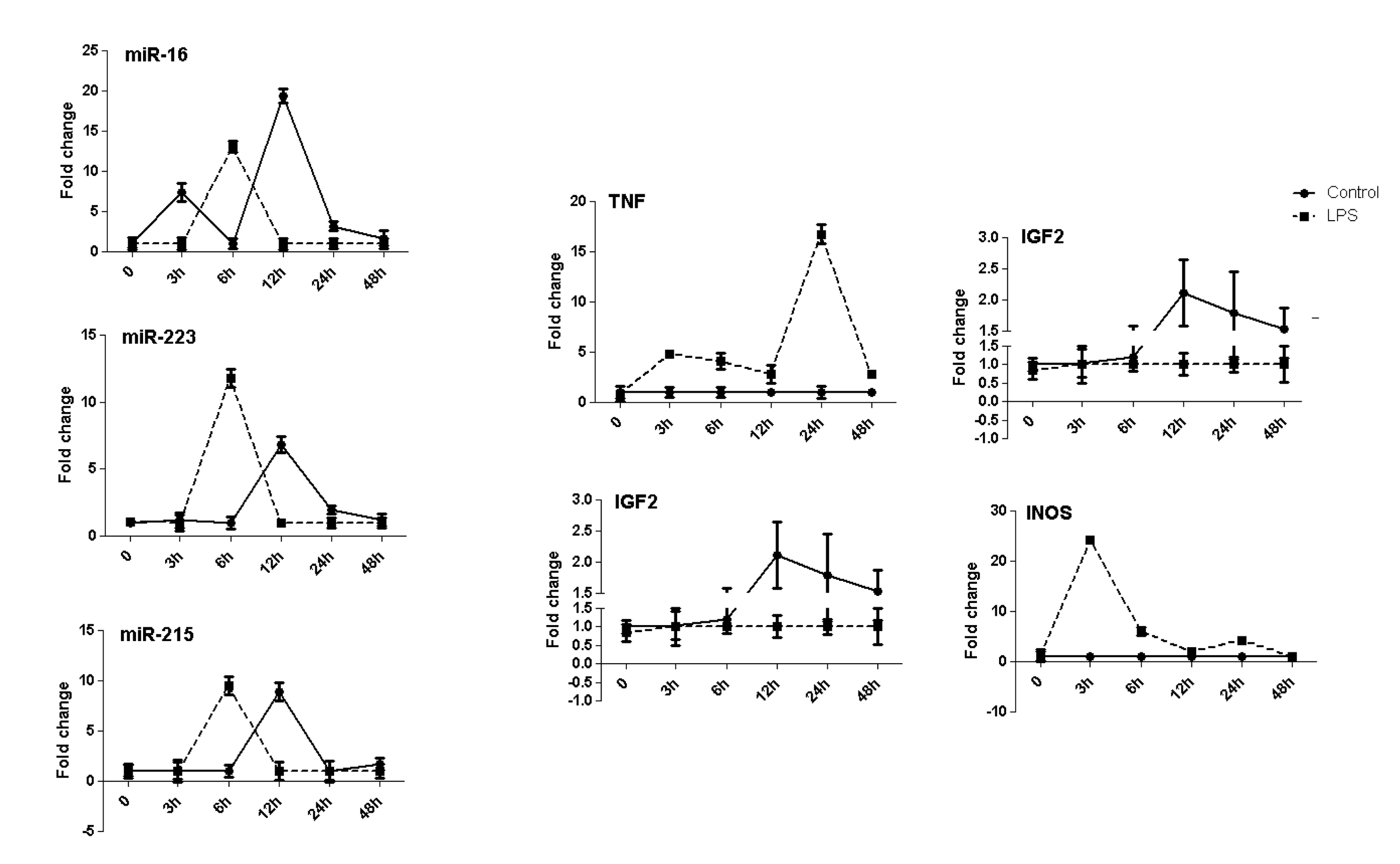

Supplement: S3 Fig — All miRNAs reached their peaks at 6h after LPS stimulation. On the other hand, some genes revealed the same trend and/or reciprocal of miRNAs. (TIF) [file pone.0119388.s003.tif]

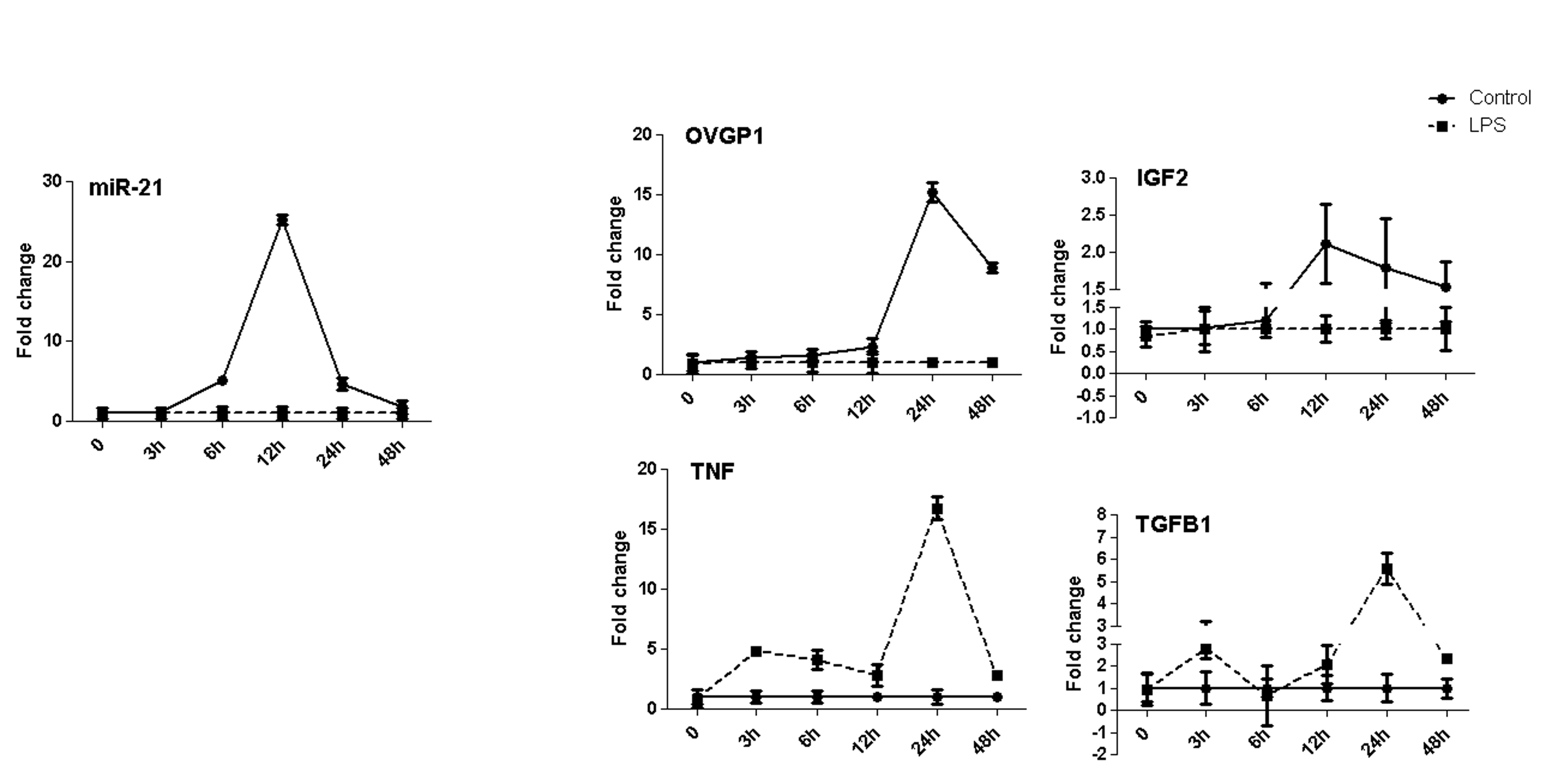

Supplement: S4 Fig — MiR-21 reached to peak at 12h then gradually reduced post LPS challenge. Both OVGP1 and IGF2 shown peaks only in untreated groups. In contrast, TNF and TGFβ1 provide clear peaks in challenged groups. (TIF) [file pone.0119388.s004.tif]

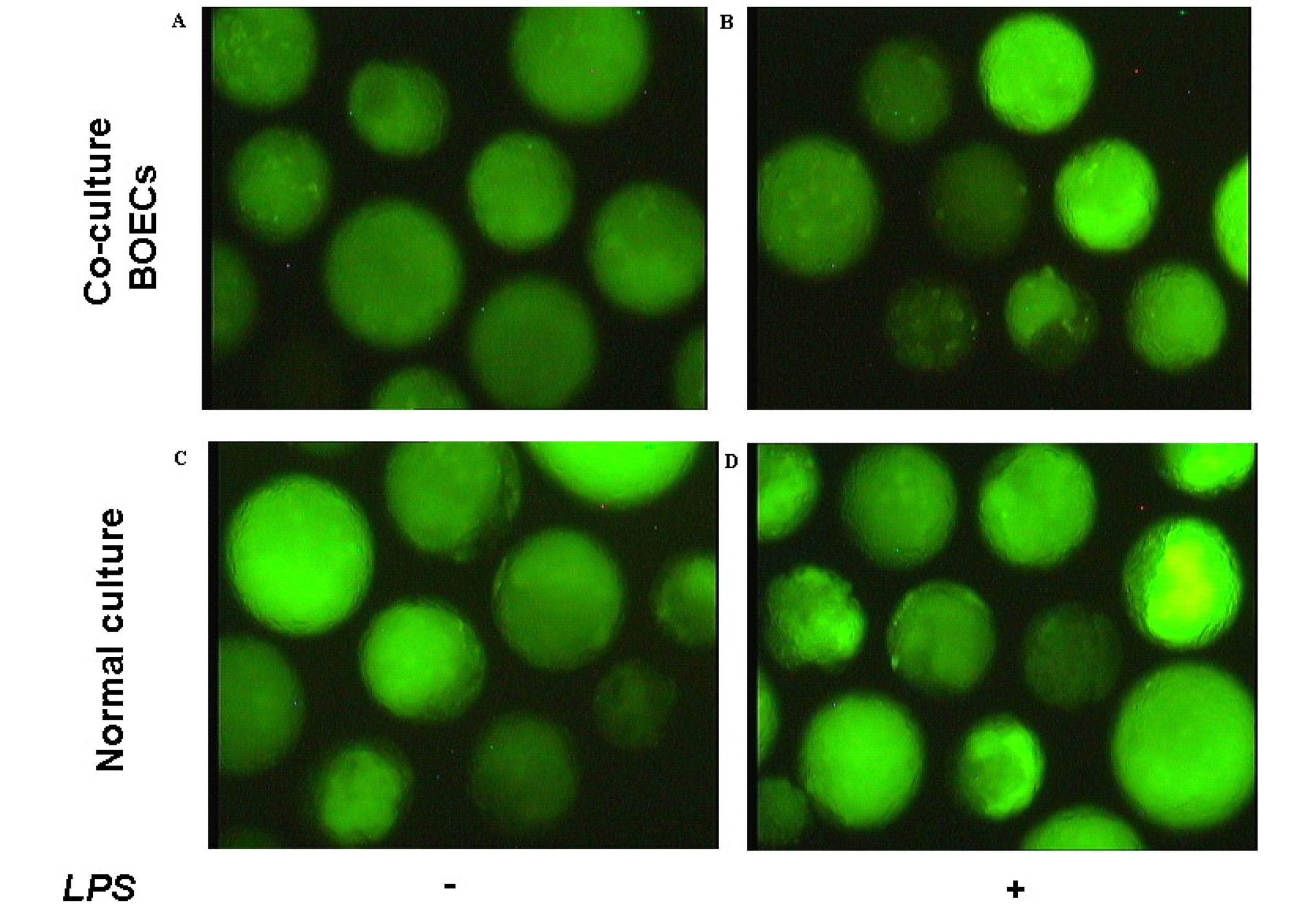

Supplement: S5 Fig — ROS production in embryo co-cultured with BOEC without or with LPS, (A & B) respectively. ROS production in bovine blastocysts which were cultured in SOF media without or with LPS, (C & D) respectively. Scale bars represent 100 μm. (TIF) [file pone.0119388.s005.tif]
